# Cyclic-di-AMP Phosphodiesterase Elicits Protective Immune Responses Against *Mycobacterium tuberculosis* H37Ra Infection in Mice

**DOI:** 10.3389/fcimb.2022.871135

**Published:** 2022-06-22

**Authors:** Yanzhi Lu, Huanhuan Ning, Jian Kang, Guangchun Bai, Lei Zhou, Yali Kang, Zhengfeng Wu, Maolin Tian, Junhao Zhao, Yueyun Ma, Yinlan Bai

**Affiliations:** ^1^ Department of Microbiology and Pathogen Biology, Basic Medical School, Air Force Medical University, Xi’an, China; ^2^ Department of Immunology and Microbial Disease, Albany Medical College, Albany, NY, United States; ^3^ Department of Clinical Laboratory, The First Affiliated Hospital, Air Force Medical University, Xi’an, China; ^4^ Department of Physiology, Basic Medical School, Ningxia Medical University, Yinchuan, China; ^5^ Student Brigade, Basic Medical School, Air Force Medical University, Xi’an, China; ^6^ Department of Clinical Laboratory, Air Force Medical Center, Air Force Medical University, Beijing, China

**Keywords:** *Mycobacterium tuberculosis*, cyclic-di-AMP, phosphodiesterase, immune response, infection, vaccine

## Abstract

Many antigens from *Mycobacterium tuberculosis (M. tuberculosis)* have been demonstrated as strong immunogens and proved to have application potential as vaccine candidate antigens. Cyclic di-AMP (c-di-AMP) as a bacterial second messenger regulates various bacterial processes as well as the host immune responses. Rv2837c, the c-di-AMP phosphodiesterase (CnpB), was found to be relative to virulence of *M. tuberculosis* and interference with host innate immune response. In this study, recombinant CnpB was administered subcutaneously to mice. We found that CnpB had strong immunogenicity and induced high levels of humoral response and lung mucosal immunity after *M. tuberculosis* intranasally infection. CnpB immunization stimulated splenocyte proliferation and the increasing number of activated NK cells but had little effects on Th1/Th2 cellular immune responses in spleens. However, CnpB induced significant Th1/Th2 cellular immune responses with a decreased number of T and B cells in the lungs, and significantly recruits of CD4^+^ and CD8^+^ T cells after *M. tuberculosis* attenuated strain H37Ra infection. Besides, we first reported that CnpB could stimulate IFN-β expression transitorily and inhibit the autophagy of macrophages *in vitro*. In mice intranasally infection model, CnpB immunization alleviated pathological changes and reduced *M. tuberculosis* H37Ra loads in the lungs. Thus, our results suggested that CnpB interferes with host innate and adaptive immune responses and confers protection against *M. tuberculosis* respiratory infection, which should be considered in vaccine development as well as a drug target.

## Introduction

Tuberculosis (TB) infection by the intracellular bacterium *Mycobacterium tuberculosis* (*M. tuberculosis*) caused 9.87 million new cases and 1.4 million deaths in 2020 (World Health Organization, 2021). Bacille Calmette-Guérin (BCG) is the only licensed vaccine against TB, but has variable efficiency in adults and could cause disseminated infection in immunocompromised individuals (Orme, 2015). Therefore, these situations make it urgently necessary to develop new, effective and safe TB vaccines.

*M. tuberculosis* possesses nearly 4 000 genes, and several immunogenic antigens from *M. tuberculosis* were explored as candidate antigens and tested under clinical investigations (Watt and Liu, 2020). These selected mycobacterial antigens included early culture filtrate proteins of *M. tuberculosis* (e.g., Ag85B, TB10.4, ESAT-6, et al.) (Ong et al., 2020), virulence-associated factors (e.g., ESAT-6, Rv2608, Rv3619c, Rv3620c, et al.) (Ong et al., 2020), and latent infection antigens (e.g., Rv2660c and Rv1813) (Wang et al., 2015). Ag85B is one of the strongest immunogens, and more than 11 vaccine candidates constructed based on Ag85B, some of which have been entered preclinical trials (Stylianou et al., 2015; Tkachuk et al., 2017; Jenum et al., 2021). Given that a variety of mechanisms involved in anti-*M. tuberculosis* immune responses, new candidate vaccine antigens should be explored based on multi-stage vaccines for better protection against *M. tuberculosis* (Kroesen et al., 2019).

Cyclic di-adenosine monophosphate (c-di-AMP) is identified as a second messenger for controlling different biological functions (He et al., 2020; Yin et al., 2020) in bacteria such as *Bacillus subtilis* (Romling, 2008), *Staphylococcus aureus* (Corrigan et al., 2011), *Streptococcus pneumonia* (Bai et al., 2013; Zarrella et al., 2020), *Mycobacterium smegmatis* (Tang et al., 2015), and *M. tuberculosis* (Corrigan and Grundling, 2013; Zhang et al., 2018). In our previous work, we reported that Rv3586, an ortholog of *B. subtilis* DisA, is the only diadenylate cyclase (DacA, later renamed as DisA) for c-di-AMP in *M. tuberculosis* (Bai et al., 2012), and could induce strong humoral immune response in mice (Cao et al., 2015). Additionally, BCG overexpressing DisA could induce stronger immune responses than BCG after *M. tuberculosis* infection (Ning et al., 2019), and provide enhanced protection against pulmonary TB in guinea pigs (Dey et al., 2020).

We also reported that Rv2837c, a DHH (Asp-His-His)-DHHA1 family protein, is one of the cyclic nucleotide phosphodiesterases (named CnpB) in *M. tuberculosis* (Yang et al., 2014), which hydrolyzes c-di-AMP into AMP in two steps (Yang et al., 2014; He et al., 2016; Wang et al., 2018). In mouse pulmonary TB model, the deletion of *cnpB* caused *M. tuberculosis* attenuated (Yang et al., 2014; Dey et al., 2017). It was reported that CnpB inhibited the innate immune cytosolic surveillance when *M. tuberculosis* resides in the cytosol through spontaneous lysis and permeable phagosomal compartment (Dey et al., 2017). CnpB-overexpressing *M. tuberculosis* strain could hydrolyze more host 2’3’-cGAMP and inhibit the STING-IRF-IFN signaling pathway than wild-type strain (Dey et al., 2017). By analyzing the transcriptomic profile of CnpB knockout *M. tuberculosis* in GSE102816 of the GEO database, we found that CnpB knockout altered gene expression involved in pathogenicity and defense response (**Figure S1**). These reports have demonstrated that CnpB is a virulence-related protein of *M. tuberculosis*, and affects bacterial virulence as well as host immune responses.

Previously, we noticed that the expression level of CnpB was much higher than DisA in *M. tuberculosis* (Bai et al., 2012; Yang et al., 2014). During the preparation of anti-CnpB immune serum, we found that anti-CnpB antibody titers were as high as diagnostic anti-Ag85B in *M. tuberculosis*-infected mice and guinea pigs. Two DHH subfamilies of *S. pneumoniae*, which also contributes to pneumococcal virulence, conferred protection against *S. pneumoniae* TIGR4 strain infection by subcutaneous inoculation (Cron et al., 2011). These results indicate that CnpB may be highly immunogenic and has a potential impact on the outcome of *M. tuberculosis* infection. In this study, we investigated CnpB protein on its immunological characteristics and its protective efficiency as subunit vaccines in *M. tuberculosis* respiratory infection model.

## Materials and Methods

### Ethics Statement

Animal studies were conducted under the approval of the Institutional Ethics Committee of Second Affiliated Hospital of Air Force Medical University, using the recommendations from the Guide for the Care and Use of Laboratory Animals of the Institute (Approval No. TDLL-2016325).

### Bacteria Strains, Cell Lines, and Animals

*M. tuberculosis* H37Ra was obtained from the National Food and Drug Administration (China). *M. tuberculosis* was grown in Middlebrook 7H9 medium (BD, USA) supplemented with 10% oleic acid-albumin-dextrose-catalase (OADC) (BD, USA) and 0.05% Tween 80, or on 7H10 agar plates (BD, USA) supplemented with 10% OADC. Murine alveolar macrophage cell line MH-S was purchased from Procell Life Science & Technology Co., Ltd. (China). Female BALB/c and C57BL/6 mice were purchased from the Animal Center of Air Force Medical University.

### Immunoinformatics Analysis of CnpB

The amino acid sequences of CnpB in *M. tuberculosis* (NP_217353) were acquired from GenBank of NCBI. IEDB (https://www.iedb.org) and BCPREDS (http://ailab-projects1.ist.psu.edu:8080/bcpred/) were used to predict the B-cell epitopes. The NetCTL (http://www.cbs.dtu.dk/services/NetCTL/) and NetMHCIIpan (http://www.cbs.dtu.dk/services/NetMHCIIpan) were used to predict the T-cell epitopes.

### CnpB Purification and Enzyme Activity Detection

Open reading frames (ORFs) of full-length CnpB (1-336 aa), DHH (29-179 aa), and DHHA1 (275-333 aa) domains of CnpB were amplified using *M. tuberculosis* genomic DNA as a template and using primers listed in **Table S1**. The DNA fragments were cloned into plasmid pET28a(+) as previously described, respectively (Pozzi et al., 2012; Yang et al., 2014; McCarthy et al., 2015). *E. coli* BL21 (DE3) strains harboring each recombinant plasmid were inoculated in LB broth, and the recombinant proteins of CnpB, DHH, and DHHA1 domains were purified using affinity chromatography according to previous work (Pozzi et al., 2012; Yang et al., 2014; McCarthy et al., 2015). Ag85B proteins were expressed and purified as previously described (Lu et al., 2018).

The enzyme activity was detected by high-performance liquid chromatography (HPLC) as previously described (Yang et al., 2014). Beforehand, reaction contained 50 mM Tris-HCl (pH 7.5), 1 mM MnCl_2_, 10 mM NaCl, 1 mM c-di-AMP (Invivogen, France), and 3 μM purified protein CnpB. The mixtures were incubated for 1 h at 37 °C, terminated by adding 1 μL 0.5 M EDTA and diluted with 40 μL ddH_2_O (Yang et al., 2014). Subsequently, 50 μL methanol was added and 20 μL of the mixture was uploaded into reverse-phase HPLC with a C18 column (250 × 4.6 mm, Vydac) as previously reported (Ryjenkov et al., 2005). Finally, different components were separated, in which nucleotides were monitored at a wavelength of 254 nm.

### Detection of Antibody Titers by ELISA

Mice sera and bronchoalveolar lavage fluids (BALFs) were collected for antibody detection using ELISA as previously reported (Ning et al., 2021). *M. tuberculosis* H37Rv infected sera samples from intravenously challenged mice and guinea pigs were obtained from our previous work (not published). TB patients sera were collected by the Tuberculosis Institute of Shannxi Province under patients’ informed consent (not published). For TB patients sera assay, the accuracy of detection was assessed by calculating the area under the receiver operating characteristic (ROC) curve. Diluted sera samples were added into CnpB, CnpB domain or Ag85B proteins coated microplates, and HRP-conjugated goat anti-mouse IgG (1:2 000, Zhongshan Co., Beijing, China) were used as the detection antibody. For immunoglobulin subclasses detection, HRP-conjugated goat anti-mouse IgG, IgG1, IgG2a, IgG2b, IgG3, IgM (1:5 000, InCellGenE LLC., Germany), or sIgA (1:2 000, Zhongshan Co., Beijing, China) were used as detection antibodies.

### Stimulation of Macrophage Cell

Bone marrow-derived macrophages (BMDMs) from female C57BL/6 mice were collected and cultured in RPMI 1640 supplemented with 15% fetal bovine serum (FBS), 100 U/mL Penicillin, 100 μg/mL Streptomycin, and 25% L929 culture supernatant. Murine alveolar macrophage MH-S and BMDM were seeded in 6-well plates at 1×10^6^ cells/well in RPMI 1640 medium supplemented with 10% FBS and incubated overnight at 37°C with 5% CO_2_. Cells were then stimulated with different concentrations of endotoxin-removed CnpB protein for the time indicated in figure legends, and medium alone was used as control. Cells were collected at time points, and total RNA was extracted using Trizol and then quantified for qRT-PCR. For Western-blot analysis, cells were lysed by RIPA buffer (Solarbio, China) supplemented with protease inhibitor cocktail (Roche, Switzerland) and phosphatase inhibitor cocktail (EpiZyme, China) for total proteins extraction at indicated time points. Anti-LC3 antibody (Sigma, USA) and anti-NF-κB (Abcam, UK) were incubated as primary antibodies, and β-actin was used as the loading control.

### Cell Transfection and Fluorescence Detection

MH-S cells were seeded at 1×10^5^ cells per well in a 24-well microplate. RFP-GFP-LC3 plasmids were transfected with lipofectamine 2000 (Invitrogen, USA) in a mass ratio of 2.5:1 without FBS for 4 h. Then the medium was replaced with a fresh medium containing 10% FBS for an additional 20 h. Cells were treated with serum-free medium or 5 μg/mL CnpB protein and examined by fluorescence microscopy. Cells were fixed with 4% paraformaldehyde, permeabilized with 0.5% Triton X-100 and blocked with 3% BSA. Cells were stained with Hoechst 33342 and observed under an Olympus fluorescence microscope.

### Survival of *M. tuberculosis* in Macrophages

1×10^5^ MH-S cells per well in 24-well microplate and 1×10^5^ BMDM cells per well in 96-well microplate were treated with CnpB for 12 h as described above. *M. tuberculosis* H37Ra was added to the cells with a multiplicity of infection (MOI) at 2:1 or 1:1 for MH-S or BMDM for 4 h. Then the extracellular bacteria were removed by washing the infected cells with sterile PBS three times, and this time point was marked as “0 h” post-infection. Macrophage cells were lysed with 0.025% SDS at indicated time points. Cell lysates were diluted and spread on 7H10 Middlebrook agar plates supplemented with OADC enrichment for bacteria colony forming units (CFU) counting, and results were presented as log_10_ CFU.

### Immunization and Infection of Mice

Mice were immunized subcutaneously with 50 μg CnpB protein in PBS mixed with or without an equal volume of incomplete Freund’s adjuvant (IFA, Sigma) for three times at 2-week intervals. The antigen dose was halved during the third immunization. An equal volume of PBS was injected into mice as naïve control. As for Ag85B, DHH, and DHHA1 proteins vaccination, the inoculation doses, and the immunization schedule were the same as that of CnpB.

Four weeks after the third vaccination, mice were challenged intranasally (i.n.) with 2.5×10^5^ CFU of *M. tuberculosis* H37Ra in 50 μL PBS. The unimmunized mice (UN group) were only infected with the same dose of *M. tuberculosis* without immunization. The naïve group of mice was treated with an equal volume of PBS.

### Detection of Complements and Cytokines Using ELISA

For quantitative determination of complements C3 and C5, mouse blood samples were collected, of which sera were separated by centrifugation at 4 °C. Sera were diluted to 1:2 500 and 1:200 for complement C3 and C5 assays respectively. Lung and spleen organ homogenates and BALF were assayed without dilution. Complement C3 and C5 were measured by the C3 ELISA kit (Alpha Diagnostic International, USA) and the C5 ELISA kit (Cloud-Clone Corp, USA) according to the manufacturer’s instructions.

For cytokines measurement, 1×10^6^ splenocytes were inoculated in 96-well plates with proteins of 5μg/mL CnpB and incubated at 37 °C with 5% CO_2_ for 72 h, then supernatants were collected for Interferon-γ (IFN-γ) and Interleukin-10 (IL-10) cytokines detection with commercial ELISA kits (Mouse ELISA, eBioscience, USA).

### Detection of Splenocytes Proliferation

Mice were sacrificed under anesthesia at 4 weeks post last vaccination or 8 weeks post-infection. Spleens were separated and single-cell splenocyte suspensions were prepared as our previous work (Ning et al., 2019). Splenocytes totalling 1×10^6^ were seeded in 96-well plates with stimulation of 5 μg/mL CnpB proteins or Ag85B proteins and incubated at 37 °C with 5% CO_2_ for 72 h. Then 20 μL MTS (CellTiter 96^®^ AQueous One Solution Cell Proliferation Assay, Promega, USA) was added and incubated for 4 h. Finally, wells were measured at the absorbance of 490 nm (A_490_) to calculate the stimulation index (SI). SI = (A_490_ of the stimulated group – A_490_ of blank control)/(A_490_ of the negative group – A_490_ of blank control).

For the CFSE assay (Lu et al., 2018), 10^7^ splenocytes were stained with 5 μM CFSE (CellTrace™ CFSE, Invitrogen, USA) at 37 °C for 20 minutes, and then stopped with 4 volumes of cold PRIM 1640 containing 10% FBS. After washing and resuspending, CFSE-labeled cells were seeded in 12-well microplates with 5 μg/well CnpB protein and incubated for 6 days. Cells were collected for flow cytometry analysis using a BD FACS Calibur cytometer. The flow cytometry data were analyzed using Modfit 5.0 software. The percentage of proliferated cells was analyzed statistically.

### qRT-PCR Analysis

After mice were sacrificed, lungs were separated aseptically and stored in RNAlater^®^ Solution (Ambion, USA). Total RNA was extracted using Trizol reagent (Ambion, USA) and then quantified. For cell assay, MH-S cells were seeded at 1 × 10^6^ cells per well in 6-well plates and cultured at 37 °C with 5% CO_2_ overnight. CnpB protein was added to the cell culture at different concentrations and cells were collected at time points. Total RNA was extracted using Trizol and then quantified as described above. qRT-PCR was carried out using primers listed in **Table S1** for detecting the transcriptional levels of cytokines by Quantitative PCR Kit (Takara, JPN) according to the instructions of the manufacturer.

### Lung Single-Cell Suspension Preparation

Four weeks post-vaccination or eight weeks post-infection, lungs were aseptically removed, cut into small pieces, and then digested in 3 mL digestion media (RPMI 1640 containing 50 μg/mL DNase I (Sigma, USA), 1 mg/mL Collagenase V (Sigma, USA), 5% fetal bovine serum, 100 U/mL Penicillin and 100 μg/mL Streptomycin (Solarbio, China) for 1 h at 37°C with 5% CO_2_. Suspensions were passed through a 70 μm cell strainer to obtain a single cell suspension. Cells were pelleted by centrifugation and lysed with red blood cell lysis buffer. After being washed with RPMI 1640 medium, cells were resuspended and adjusted to a suitable density in complete RPMI 1640 medium.

### Flow Cytometry Analysis

Four weeks post-vaccination or eight weeks post-infection, single-cell suspensions of the spleen and lung were prepared according to our previous work (Ning et al., 2019). For different immune cell subsets analysis, 1.5×10^6^ cells were stained with Live/Dead Zombie NIR dye (BioLegend, USA). Fc receptors were blocked by anti-mouse CD16/32 (BioLegend, USA). Lung cells were stained with antibodies of BV510-anti-CD3, PE/Cy7-anti-CD4, PE-anti-CD8, FITC-anti-Ly6G, BV421-anti-CD19, PerCP-anti-CD49b, Alexa Fluor 700-anti-CD11b, and Alexa Fluor 647-anti-F4/80 (BioLegend, USA) for 30 min on ice and in darkness. Spleen cells were stained with BV510-anti-CD3, Alexa Fluor 700-anti-CD11b, Alexa Fluor 647-anti-F4/80, PE/Cy7-anti-CD69, PerCP-anti-CD49b, FITC-anti-CD19, PE/Cy7-anti-MHC II, PerCP-anti-CD86, and FITC-anti-CD80 (Abcam, UK).

For cytokines staining of T cells, 3×10^6^ splenocytes were seeded in 24-well microplates with 5 μg/mL CnpB proteins and incubated at 37 °C for 48 h (Lu et al., 2018). Protein transport inhibitor of Brefeldin A Solution (BioLegend, USA) was added for 12 h. Surface markers of PerCP-anti-CD4 and PE-anti-CD8 were stained as described above. Then, cells were permeabilized using Fixation/Permeabilization Kit (BD, USA), followed by cytokines staining of FITC-anti-IFN-γ, and APC-anti-IL-10 (BioLegend, USA). Cells were analyzed by flow cytometer (BD FACS Canto), and data were analyzed with FlowJo software (Treestar, USA).

### Immunohistochemistry and CFU Enumeration

Lung tissues of infected mice were fixed in formalin, then embedded in paraffin, and sectioned. For immunohistochemistry, anti-CD4 and CD8 antibodies (Abcam, UK) were used for the immunohistochemistry assay. The integrated optical density of CD4 or CD8 in each immunohistochemistry slide was calculated by Image-Pro Plus 6.0 software. For pathological analysis, lung tissue sections were stained with hematoxylin-eosin (HE). All the sections were observed under an optical microscope. The pathological changes (e.g., peribronchiolitis, perivasculitis, alveolitis, and granuloma formation) of the lung were scored as 0, 1, 2, 3, 4, or 5 for absent, minimal, slight, moderate, marked, or strong changes respectively, according to Dormans’ report (Dormans et al., 2004).

For CFU counting, lungs and spleens were homogenized, and homogenates were diluted and plated on 7H10 Middlebrook agar plates. Plates were incubated at 37 °C until colonies were visible. CFUs were counted and the results were expressed as log_10_CFU per lung or per spleen.

### Statistical Analysis

Statistical analysis was performed using the software GraphPad Prism 5.0. One-Way ANOVA was performed for comparison of groups over two. Two-Way ANOVA was performed for comparison of groups in each treatment. T-test was performed for two groups comparison. *P* < 0.05 was considered a statistically significant difference. The data of the ROC curve were generated by SPSS Statistics 25.0.

## Results

### Antigenic Prediction and Anti-CnpB Antibody Levels Showed Immunogenicity of CnpB

Since the deletion of CnpB led to reduced virulence of *M. tuberculosis* (Yang et al., 2014; Dey et al., 2017), CnpB could be strongly taken considered as a prophylactic candidate vaccine antigen against *M. tuberculosis*. The prediction of allergen and B-cell and T-cell epitopes demonstrated that CnpB possesses antigenic epitopes (**Figure 2A** and **Tables S2**, **S3**), implying CnpB could be a potential antigen for TB vaccines like Ag85B and ESAT-6.

**Figure 1:**
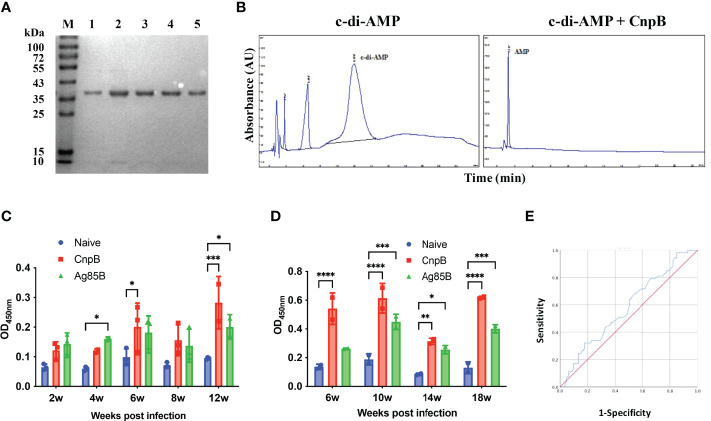
Characterization of recombinant CnpB and detection of humoral immune response against CnpB. **(A)** CnpB was purified by Ni-NTA. Eluted proteins were analyzed using SDS-PAGE. Lane M, molecular marker. Lanes 1 to 5: eluted samples. **(B)** CnpB hydrolyzes c-di-AMP to AMP detected by HPLC. **(C)** After the mice were infected with *M. tuberculosis*, ELISA was used to detect the levels of anti-CnpB and anti-Ag85B antibodies in sera (*n*=3). **(D)** After the guinea pigs were infected with *M. tuberculosis*, ELISA was used to detect the levels of anti-CnpB and anti-Ag85B antibodies in sera (*n*=2). The results were shown as mean ± SD. **(E)** The receiver operating characteristic (ROC) curve of antibodies in sera from TB patients (blue curve). The X-axis represents the false positive rate, and the Y-axis represents the true positive rate. *P < 0.05, **P < 0.01, ***P < 0.001, ****P < 0.0001.

As is known, Ag85B is one of the strongest antigens of *M. tuberculosis*, and novel fusion proteins with Ag85B have entered clinical trials as TB vaccine candidates (Suliman et al., 2019). In this study, recombinant CnpB was purified by affinity chromatography as we previously reported (Yang et al., 2014) (**Figure 1A**). In addition, the phosphodiesterase activity of CnpB hydrolyzing c-di-AMP was confirmed by HPLC (**Figure 1B**), which was consistent with our previous report (Yang et al., 2014). We found that the levels of anti-CnpB antibodies in sera of *M. tuberculosis* infected mice were as high as those of anti-Ag85B, and gradually increased with the infection time and reached the highest level at 12 weeks post-infection (**Figure 1C**). Similarly, the levels of anti-CnpB antibodies in *M. tuberculosis* infected guinea pigs were even higher than those of anti-Ag85B antibodies (**Figure 1D**). In sera of TB patients, the levels of anti-CnpB antibodies were also elevated. The area under the receiver operating characteristic (ROC) curve (AUC) of CnpB was 0.58 (**Figure 1E**) which was not comparable with two classic antigens of *M. tuberculosis* CFP-10 and ESAT-6 (Luo et al., 2017). These data suggested that CnpB exhibited strong immunogenicity, but showed weak diagnostic potential.

### CnpB Induced Strong Humoral Response in Sera and Mucosa

**Figure 2:**
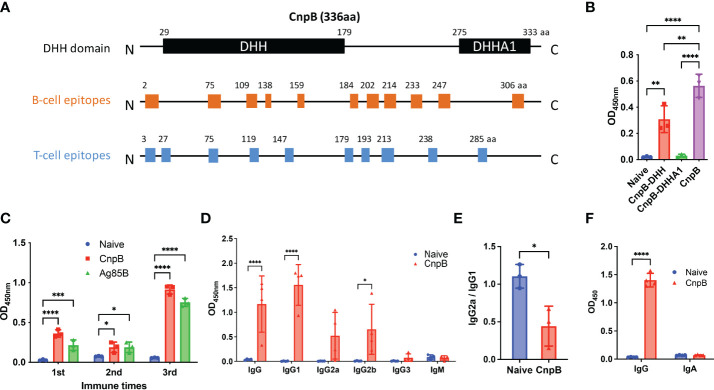
The humoral immune response of CnpB immunization in mice. **(A)** Prediction of B-cell and T-cell epitopes on CnpB. **(B)** Sera antibodies of DHH, DHHA1 truncated proteins, and CnpB full-length protein were detected by ELISA in CnpB-immunized mice (*n*=3). **(C)** Antigen-specific antibodies against CnpB or Ag85B in sera from CnpB or Ag85B immunized mice (50 μg of CnpB, DHH (1-179aa), DHHA1 (180-336aa) or Ag85B protein were inoculated subcutaneously with incomplete Freund’s adjuvant for three times at 2-week intervals) detected by ELISA (*n*=3). **(D)** Antibody subclasses against CnpB in sera of CnpB immunized mice detected by ELISA (*n*=4). **(E)** IgG2a/IgG1 ratio of sera antibody subclasses in CnpB immunized mice (*n*=3). **(F)** The levels of IgG and sIgA in bronchoalveolar lavage fluid of CnpB immunized mice (*n*=4). The results are expressed as mean ± SD. *P < 0.05, **P < 0.01, ***P < 0.001, ****P < 0.0001.

CnpB of *M. tuberculosis* is a DHH-DHHA1 domain protein as Pde2, a c-di-AMP phosphodiesterase in *Streptococcus pneumoniae* (Bai et al., 2013). The N-terminal DHH and C-terminal DHHA1 domains consist of residues 29-179 aa and 275–333 aa, respectively (Yang et al., 2014). The DHH domain, having a five-parallel strand β-sheet that is sandwiched by 10 α-helices, has the phosphodiesterase activity catalytic core (Srivastav et al., 2014). The DHHA1 domain contributes to both the recognition and stabilization of substrates (He et al., 2016). By immunoinformatics analysis, we found that most B-cell and T-cell epitopes were distributed in the DHH domain and spacer sequence instead of the DHHA1 domain (**Figure 2A**). Then, two truncated proteins of DHH and DHHA1 were expressed and purified for immunogenicity evaluation in mice. It was shown that the DHH domain dominated the major immunogenicity of CnpB rather than the DHHA1 domain (**Figure 2B**), which was consistent with the epitopes prediction of CnpB (**Figure 2A**). These data suggested the immune response was mainly derived from the DHH domain and the spacer sequence.

Next, we evaluated the humoral immune response induced by full-length CnpB subcutaneous vaccination. The levels of anti-CnpB antibody in sera rose sharply and reach a plateau 4 weeks post 3rd immunization (**Figure 2C**). In sera of immunized mice, anti-CnpB and anti-Ag85B antibodies had comparable OD_450_ detected by the same dose of antigens (10 μg/mL) at 1 : 6 400 dilution in ELISA assay. Meanwhile, the antibody titers of anti-CnpB and anti-Ag85B were both 1 : 102 400 (the ratio of OD_sample_/OD_negative control_ was over 2.1) by serial dilutions. Therefore, we believe that CnpB is able to induce comparable antibody titers with Ag85B. IgG1 was the main antibody subclass (**Figure 2D**), and the ratio of IgG2a/IgG1 was significantly decreased in the CnpB group (**Figure 2E**), which represents a reduced Th1 balance (Paydarnia et al., 2020). Meanwhile, IgG levels in bronchoalveolar lavage fluid (BALF) were significantly elevated in CnpB subcutaneously immunized mice (**Figure 2F**). These data suggested that CnpB could trigger an antigen-specific humoral response at both systemic and local levels by subcutaneous inoculation.

### CnpB Had Different Effects on Immune Response of Spleen and Lung

**Figure 3:**
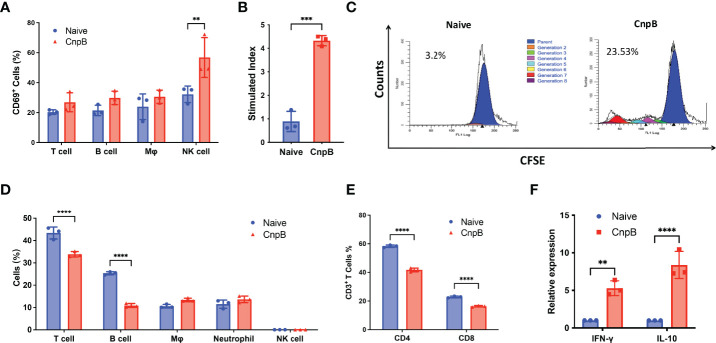
Detection of cellular immune response in CnpB-immunized mice. **(A)** Proportions of immune cells in the spleens of CnpB immunized and naïve mice (*n*=3). **(B)** The proliferation levels of splenocytes from CnpB immunized mice stimulated by PPD protein. Splenocytes were isolated from mice 4 weeks after the last CnpB immunization and analyzed for proliferation using an MTS assay (*n*=3). **(C)** CFSE proliferation assay of splenocytes from CnpB immunized mice stimulated by CnpB. Flow cytometry was used to detect the proliferation of CFSE-labelled splenocytes, which were stimulated by 5 μg/mL CnpB proteins. The percentage numbers indicated the proportion of proliferating cells in total cells (*n*=3). **(D)** The percentage of T cells, B cells, macrophages, neutrophils, and NK cells in the lungs of CnpB immunized mice (*n*=3). **(E)** Percentage of CD4^+^ and CD8^+^ T cells in the lungs of CnpB immunized mice. (*n*=3). **(F)** qRT-PCR was used to determine the relative transcriptional levels of Th1/Th2 cytokines, including IFN-γ and IL-10, in lung tissue of CnpB immunized mice (*n*=3). The results are expressed as the mean ± SD. **P < 0.01, ***P < 0.001, ****P < 0.0001.

Cellular immunity is important for protection against intracellular bacterium such as *M. tuberculosis*. CD69 is the early leukocyte activation antigen and is mainly expressed by activated T cells, B cells, and natural killer (NK) cells (Lauzurica et al., 2000). For splenocytes, CnpB stimulation *in vitro* up-regulated CD69 expression in NK cells, but not in T cells, B cells, and macrophages (**Figure 3A**). Besides, the expressions of the costimulatory molecules CD80/CD86 and MHC-II in macrophages were comparable between CnpB immunized and naïve mice (**Figure S2A**), implying that CnpB immunization had little effect on the antigen presentation of macrophages.

Splenocytes’ proliferative response is one of the indicators of cellular immune response in the host. Through MTS and CFSE assay, we found that CnpB immunization stimulated significant splenocyte proliferation (**Figures 3B, C**). Th1/Th2 immune responses play important roles in the protection against *M. tuberculosis* in the host (Abebe, 2019). T cells from PPD-positive TB patients could produce both IFN-γ and IL-10, and the proliferation levels of Th1 and Th2 types T cells were elevated (Harling et al., 2019). However, CnpB immunized mice failed to induce IFN-γ and IL-10 release from splenocytes (**Figure S2B**). Neither, CnpB vaccination didn’t alter the proportions of CD4^+^ and CD8^+^ T cells in the spleen (**Figure S2C**), and the percentages of CD4^+^ T cells secreting Th1/Th2 cytokines were not affected by CnpB immunization (**Figure S2D**). These indicated that CnpB immunization could induce splenocyte proliferation, but did not stimulate T cell activation, corresponding to the ratio of IgG2a/IgG1 (**Figure 2E**).

Next, we investigated the immune cell population in the lungs after CnpB inoculation. CnpB inoculation did not change the proportions of NK, macrophages and neutrophils (**Figure 3D**). To our surprise, CnpB immunization decreased the proportions of T and B cells in the lungs (**Figure 3D**). The proportions of CD4^+^ and CD8^+^ T cells, the main cellular immune cells against *M. tuberculosis* and other pathogens, were also reduced by CnpB immunization (**Figure 3E**). On the contrary, CnpB immunization up-regulated transcription of IFN-γ and IL-10 in the lungs (**Figure 3F**). These results suggested that CnpB may have different effects on cellular immunity in spleens and lungs, as well as innate immunity.

### CnpB Inhibited Autophagy Temporarily in Lung Macrophages

**Figure 4:**
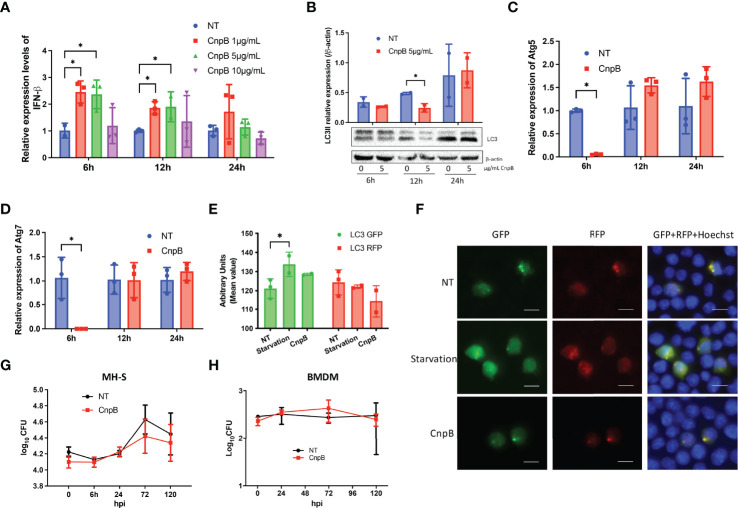
Type I IFN and autophagy responses stimulated by CnpB in macrophages. **(A)** In MH-S cells, IFN-β transcriptional levels were analyzed by qRT-PCR at 6 h, 12 h, and 24 h post-stimulation with different doses of CnpB (*n*=3). NT, no treated. **(B)** LC3-I/II protein levels were analyzed by Western-blot post-stimulation with different doses of CnpB (*n*=2). NT, no treated. The figure shown is a representative of two repeat experiments. **(C**, **D)** Atg5 and Atg7 transcriptional levels were analyzed by qRT-PCR at 6 h, 12 h, and 24 h post 5 μg/mL CnpB stimulation (*n*=3). **(E**, **F)** MH-S cells transfected with RFP-GFP-LC3 plasmids and LC3 puncta were analyzed by immunofluorescence post-CnpB stimulation for 4 h. Semi-quantitative analysis of panel **(F)** showed in panel **(E)**. RFP, red fluorescence protein. GFP, green fluorescence protein. Scale bar is 50 μm (*n*=3). **(G, H)** MH-S cells in 24-well microplates **(G)** and BMDM cells in 96-well microplates **(H)** were treated with 5 μg/mL CnpB for 12 h, then cells were infected with *M. tuberculosis* H37Ra at MOI of 1 (*n*=2 or 3). Bacteria were removed 4 h post-infection, this time point is termed as “0 h”. Bacteria CFUs were determined at indicated time points post-infection. The results are expressed as mean ± SD. *P < 0.05.

CnpB deleted *M. tuberculosis* strain induced STING-dependent pathway activation and type I interferon response in macrophages (Dey et al., 2017). Further, we investigated whether CnpB could affect the innate immune response of MH-S, a cell line of lung macrophages. IFN-β mRNA was significantly elevated after 6 h, lasting for 12 h by CnpB stimulation in MH-S, and returned to normal 24 h after treatment (**Figure 4A**). However, a high dose of CnpB (10 μg/mL) did not interfere with IFN-β transcription (**Figure 4A**). *M. tuberculosis*-derived c-di-AMP, a substrate of CnpB, induced significant IFN-β expression through activating STING in macrophages (Yang et al., 2014; Dey et al., 2017; Ning et al., 2019). However, in this study there was no infection in CnpB-treated macrophages, suggesting activation of the STING pathway is not mediated by c-di-AMP. Further, we found that CnpB did not affect the expression of NF-κB (**Figure S3**), inferring regulation of IFN-β expression was independent of the STING/IKK/NF-kB pathway. In MH-S cells, a low dose of CnpB induced type I interferon response at a short time, but both low and high doses had little effect at a long time, also suggesting that CnpB may act on other innate immune mechanisms of macrophages.

Dey et al. (Dey et al., 2015) showed that overexpression of *disA* in *M. tuberculosis* to secrete excessive c-di-AMP enhanced the production of IFN-β and increased macrophages autophagy, and this strain showed reduced virulence in mice. However, it was reported that CnpB inhibited the innate immune cytosolic surveillance such as cGAS-cGAMP pathway in macrophages (Dey et al., 2017), and autophagy is generally believed to help fight against infection (Dey et al., 2015). Western-blot analysis revealed that the LC3-II levels of macrophages was significantly reduced at 12 h post-low-dose CnpB stimulation, and returned to normal 24 h post-stimulation (**Figure 4B**). The transcriptional levels of Atg5 and Atg7, the key molecules in the initial stage of autophagy, were drastically down-regulated 6 h post-CnpB stimulation (**Figures 4C, D**) and back to normal levels 12 h post-stimulation, in line with the reduced levels of downstream LC3-II at 12 h post-stimulation. In addition, MH-S cells were transfected with RFP-GFP-LC3 plasmid, followed by CnpB protein stimulation for 4 h, and cultured for 24 h. The GFP fluorescence showed a trend of inhibition with CnpB pre-treatment, but not the red fluorescent particles (**Figures 4E, F**). These results implied that CnpB negatively affected the autophagy of macrophages in a short time, and the reduced autophagy might negatively activate STING, thereby raising the expression of IFN-β in a short time (**Figure 4A**).

Further, MH-S and BMDM were treated with 5 μg/mL CnpB for 12 h and infected with *M. tuberculosis* H37Ra (MOI=1). After 4 h infection, *M. tuberculosis* CFUs showed no significant difference between the negative control and CnpB treatment group in MH-S (**Figure 4G**) and BMDM cells (**Figure 4H**). Besides, *M. tuberculosis* H37Ra was added to infect MH-S with a multiplicity of infection (MOI) at 2:1 (**Figure S4**). The untreated cells showed a similar replication of *M. tuberculosis* as the CnpB group did, and no replication with time, suggesting that higher MOI may be the reason to restrict the replication in MH-S. In addition, CnpB treatment did not change the survival of *M. tuberculosis* in macrophages, which might be the combined results of IFN-β upregulation (**Figure 4A**) and autophagy inhibition (**Figure 4F**), suggesting innate immune response induced by recombinant CnpB protein is insufficient to restrict bacterial survival in macrophages.

### CnpB Induced Humoral and Lung Mucosal Immunity in *M. tuberculosis* H37Ra Intranasally Infected Mice

**Figure 5:**
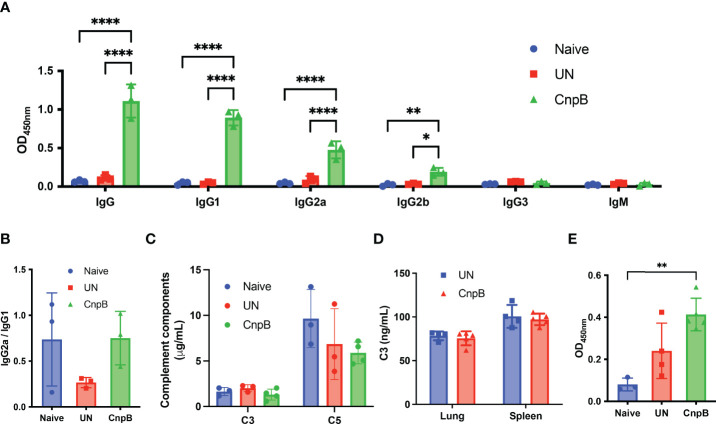
Antibody and complement detection of CnpB-immunized mice after *M. tuberculosis* intranasal infection. After *M. tuberculosis* H37Ra intranasal challenge for 8 weeks, antibodies and complements were detected. **(A)** Sera of CnpB immunized (CnpB) mice and unimmunized (UN) mice were collected for antibodies detection using ELISA (*n*=3). The collection time points was 8 weeks after infection. **(B)** The ratio of IgG2a/IgG1 in panel A was calculated (*n*=3). **(C, D)** The levels of complement C3/C5 in sera (*n* = 3) **(C)** and complement C3 in lung/spleen homogenate (*n* = 4) **(D)** were assayed by ELISA. **(E)** The levels of sIgA in BALF were assayed by ELISA. The results are shown as mean ± SD. *P < 0.05, **P < 0.01, ****P < 0.0001.

Since that *M. tuberculosis* attenuated strain H37Ra and virulent strain H37Rv was capable to induce comparable levels of circulating IgG (Ning et al., 2017), we used H37Ra instead of H37Rv. Eight weeks after *M. tuberculosis* nasal challenge, anti-CnpB IgG, IgG1, IgG2a, and IgG2b increased significantly in sera of immunized mice (**Figure 5A**). The ratio of IgG2a/IgG1 showed an increasing tendency, implying Th1 response was induced (Mansury et al., 2019) (**Figure 5B**), which was different from previous results without infection (**Figure 2E**), suggesting CnpB immunization might reverse the IgG2a/IgG1 balance after *M. tuberculosis* infection.

Previous studies have reported that patients with active pulmonary TB elicited the elevation of levels of IgG1 and IgG3 subclasses in sera (Correa et al., 2019), which played important roles in complement activation (Jovic and Cymer, 2019). We found that the levels of complement C3 and C5 decreased slightly in sera of CnpB immunized mice (**Figure 5C**), but levels of complement C3 in homogenates of lungs and spleens remained unchanged (**Figure 5D**), suggesting that high levels of IgG subclasses might activate and deplete complement system through the classical pathway. Meanwhile, although CnpB subcutaneous immunization did not induce sIgA production (**Figure 2F**), *M. tuberculosis* intranasal challenge caused a significant increase of anti-CnpB sIgA secretion in BALF of CnpB immunized mice (**Figure 5E**). Thus, CnpB subcutaneous immunization could induce a significant mucosal immune response when infected with *M. tuberculosis* by the respiratory tract.

### CnpB Vaccination Induced Enhanced Cellular Immune Responses after Intranasal Infection of *M. tuberculosis* H37Ra

**Figure 6:**
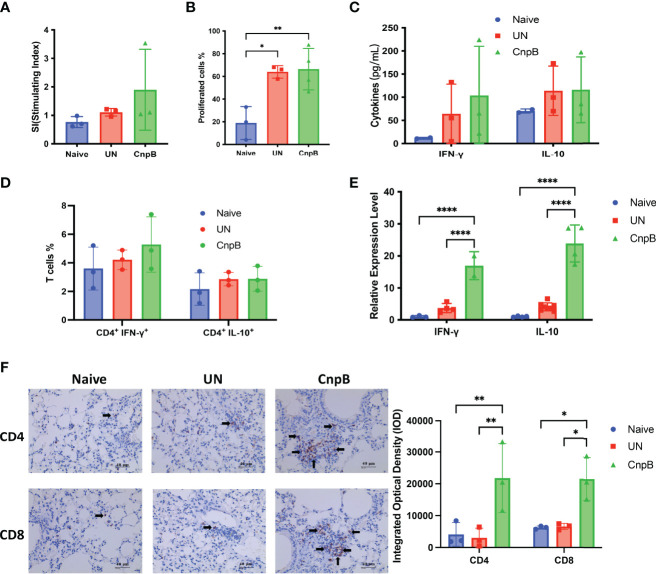
Cellular immune responses of CnpB-immunized mice after *M. tuberculosis* intranasal infection. After *M. tuberculosis* H37Ra intranasal challenge for 8 weeks, cellular immune responses were determined as follows. **(A)** Proliferation of splenocytes measured by MTS reagent (*n*=3). **(B)** Splenocytes were labeled with CFSE reagent, and then stimulated with 5 μg/mL CnpB proteins. Proportion of proliferated cells was assayed by flow cytometry (*n* = 3 or 4). **(C)** Levels of cytokines IFN-γ and IL-10 secreted by splenocytes were detected using ELISA (*n* = 2 or 3). **(D)** Splenocytes were stimulated by 5 μg/mL CnpB proteins, and percentages of CD4^+^ T cells producing IFN-γ and IL-10 were determined by flow cytometry (*n*=3). **(E)** Transcriptional levels of IFN-γ and IL-10 cytokines in the lungs were detected using qRT-PCR (*n* ranges from 2 to 8). **(F)** Immunohistochemical observation and integrated optical density of CD4^+^ and CD8^+^ T cells in lung tissues of CnpB-immunized mice infected with H37Ra intranasally (*n*=3). The arrows represented the stained CD4^+^ and CD8^+^ T cells. The results are expressed as mean ± SD. *P < 0.05, **P < 0.01, ****P < 0.0001.

It is known that *M. tuberculosis* infection could suppress cellular immune response through multiple mechanisms for its survival (Both et al., 2018). The splenocyte proliferation was not changed in CnpB immunized mice compared to *M. tuberculosis* intranasal infection mice (**Figures 6A, B**). Meantime, the levels of secreted IFN-γ and IL-10 were not significantly changed compared with unimmunized mice (**Figure 6C**). As well, the Th1 (CD4^+^ IFN-γ^+^) and Th2 (CD4^+^ IL-10^+^) cells proportions were not significantly altered compared to naïve mice (**Figure 6D**), indicating that CnpB immunization might not stimulate cellular immune responses in spleens after *M. tuberculosis* intranasal infection.

However, in the total cells of the lungs, the transcriptional levels of IFN-γ and IL-10 (Th1/Th2 cytokines) of CnpB immunized mice were significantly higher than those of unimmunized mice after *M. tuberculosis* infection (**Figure 6E**). The immunohistochemistry showed that CnpB immunization could recruit CD4^+^ and CD8^+^ T cells to the interstitium of the lung after *M. tuberculosis* infection (**Figure 6F**), corresponding to the results of increased IgG2a/IgG1 ratio reflecting Th1 balance (**Figure 5B**). These results implied that CnpB immunization mainly induced the cellular immune response in lungs rather than in spleens after *M. tuberculosis* infection, and CnpB might perform a different immune mechanism compared to other vaccine antigens such as Ag85B.

### CnpB Immunization Reduced the Inflammation and Host Susceptibility to *M. tuberculosis* H37Ra Respiratory Infection

**Figure 7:**
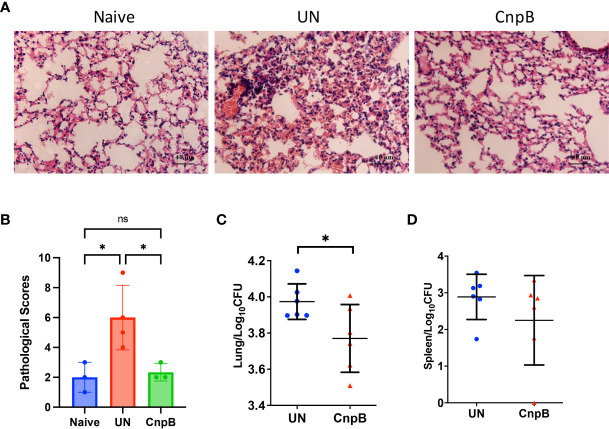
Pathological changes and bacterial loads in CnpB immunized mice after *M. tuberculosis* intranasal infection. **(A, B)** Pathological observation **(A)** and scores **(B)** of lungs of CnpB-immunized mice with *M. tuberculosis* H37Ra intranasal infection (*n* = 3 or 4). **(C, D)** Bacterial loads in the lungs **(C)** and spleens **(D)** of CnpB-immunized mice after *M. tuberculosis* H37Ra intranasal infection (*n*=6), respectively. The results are expressed as mean ± SD. *P < 0.05, ns indicates no significance.

Inflammation is a basic pathological process during *M. tuberculosis* infection. After *M. tuberculosis* infection, the spleens of mice were enlarged by naked eyes, but not alleviated in CnpB immunized mice. However, in the lungs HE stained sections, inflammatory cell infiltration was significantly reduced in CnpB immunized mice, and the alveolar structure intact was maintained compared to those of unimmunized mice (**Figures 7A, B**). Compared to unimmunized mice, the *M. tuberculosis* CFUs in lungs decreased significantly in CnpB immunized mice infected with *M. tuberculosis* (**Figure 7C**), while the CFUs in spleens showed no significant difference (**Figure 7D**) (*n* = 6). These results were consistent with the results that CnpB induced cellular immune response in lungs rather than in spleens, implying CnpB performed effective protection against *M. tuberculosis* respiratory tract infection.

## Discussion

Previous studies have reported that the DHH/DHHA1 domain family proteins in bacteria can be divided into two subfamilies (Wang et al., 2018). The first subfamily contains membrane-bound proteins, mainly existed in microorganisms like *Bacillus subtilis*, *Streptococcus pneumoniae*, *Listeria monocytogenes*, and *Staphylococcus aureus* (Wang et al., 2018). The second subfamily includes stand-alone DHH/DHHA1 phosphodiesterases like CnpB, which have been reported to exist in *M. tuberculosis* (Wang et al., 2018) and other mycobacteria like *Mycobacterium smegmatis* (Srivastav et al., 2014; Tang et al., 2015). Previous work including ours found that CnpB deleted *M. tuberculosis* strain showed attenuated virulence in mice (Yang et al., 2014; Dey et al., 2017). During our study, we noticed that anti-CnpB antibodies were as high as that of Ag85B (**Figure 1C**). Further, protein epitope analysis showed T and B cells epitopes distributed in the whole CnpB sequence (**Figure 2A**). It was reported that Rv0159c, an intracellular protein, could induce protection against *M. tuberculosis* and had been explored as a subunit vaccine (Singh et al., 2013). Dey et al. (Dey et al., 2017) reported that CnpB is distributed on the cell membrane and in the cytoplasm of *M. tuberculosis*. Thus, these results suggested that CnpB as a cytoplasmic protein might be released from bacteria and then presented by host cells to induce immune response, which might inhibit bacterial survival.

Recently, more and more evidence has shown that antibodies have protective effects (Rijnink et al., 2021). Targets and structural or functional differences of specific antibodies have been observed during different TB disease states (Li et al., 2017; Lu et al., 2019). The spleen is the largest peripheral immune organ of the host containing abundant T and B lymphocytes, which exerts immune recognition and clearance of the foreign pathogens in the circulation (Chotivanich et al., 2002). Thus, one indicator for evacuating systemic dissemination of *M. tuberculosis* is bacterial loads in the spleen (Krishnan et al., 2010), which is controlled by circulating IgG and systemic cellular immune responses. We observed no significant CFU reduction in the spleen of CnpB-immunized mice compared to UN mice, but several mice had extremely low CFU in the spleen (**Figure 7D**), suggesting that the IgG in sera slightly affected the systemic spread of *M. tuberculosis*. For mucosal immune response, studies have demonstrated that human sIgA treatment reduced the *M. tuberculosis* CFU in the lungs of *M. tuberculosis* infected mice (Alvarez et al., 2013), and secretion of sIgA also positively correlated with the protective immune response against *M. tuberculosis* (Ai et al., 2013). We found that subcutaneous immunization of CnpB induced the production of sIgA in mucosa of respiratory tract after *M. tuberculosis* intranasal infection, which may partly explain the protection of CnpB subcutaneous immunization against pulmonary *M. tuberculosis* infection.

Although anti-CnpB antibody levels in *M. tuberculosis* infected mice were extremely high, serum anti-CnpB antibody might not be used as a biomarker of *M. tuberculosis* infection for diagnosis, which was confirmed by the ROC curve. This suggested that the specificity of CnpB induction antibodies might be elevated in TB patients, but were not predominant. Moreover, specific antibodies against *M. tuberculosis* produced by the host tend to be used as markers reflecting protective immune responses (Rijnink et al., 2021) rather than diagnostic biomarkers. Anti-CnpB IgG1, IgG2a, and IgG2b were the main subclasses of IgG (**Figures 3A**, **5A**) and might be involved in the induction of downstream pro-inflammatory responses and complement activation. C3 and C5 in sera showed a downward trend after *M. tuberculosis* infection, implying that complement may be activated to a certain extent (Jagatia and Tsolaki, 2021).

Cellular immunity is the key for host to defend against *M. tuberculosis* infection, in which evaluation of systemic cellular immune response can be achieved by detecting indicators of splenocytes (Lee et al., 2013). In this study, we found that the spleen was not significantly enlarged after CnpB immunization, but it was enlarged after *M*. *tuberculosis* infection. Rapid expression of CD69 in splenocytes of CnpB immunized mice, as a costimulatory signal and marker molecule, enhances the cellular activation, proliferation, and differentiation of NK cells except T cells (**Figure 3D**). Meanwhile, the expression of costimulatory molecules CD80/CD86 and MHC II of macrophages were not affected significantly by CnpB (**Figure S2A**). Subsequently, before or after *M. tuberculosis* infection, CnpB immunization enhanced splenocyte proliferation but not stimulated the production of IFN-γ, suggesting its limited capability of inducing a systemic cellular immune response in this study. Th1 and Th2 cellular immune response play important roles in the host protection against tuberculosis (Abebe, 2019), but CnpB had little effect on the splenocytes’ cellular immunity, which might be one of the reasons that CnpB immunization could not reduce the *M. tuberculosis* loads in spleens (**Figure 7D**).

Previous studies reported that CD4^+^ T cells play a central role in protective immunity against *M. tuberculosis* infection through assisting B or cytotoxic T cells and releasing cytokines (Sallusto, 2016; Abebe, 2019). Although CnpB immunization induced down-regulation of the proportion of CD4^+^ and CD8^+^ T cells in the lungs (**Figure 3E**), it raised the transcriptional level of IFN-γ (**Figure 3F**) without altering the number of macrophages in the lungs (**Figure 3D**), indicating that CnpB elicits a complex cellular immune response in lungs. However, after *M. tuberculosis* infection, CnpB immunization induced T cells to accumulate in the alveolar interstitium (**Figure 6F**) and raised the transcriptional level of IFN-γ. The reason might be that *M. tuberculosis* infection enhanced the immune function of CnpB resulting in increased cellular immune response, which contributed to the reduction of *M. tuberculosis* CFU in the lungs of CnpB subcutaneous immunization mice (**Figure 7C**). Moreover, our previous work found that c-di-AMP adjuvanted ESAT-6 intranasal immunization in mice induced decresed proportions of CD8^+^ rather than CD4^+^ T cells, which provided protection against *M. tuberculosis* H37Ra infection (Ning et al., 2021). However, the reason for the reduction of pulmonary immune cells in this study still needed to be further explored.

C-di-AMP secreted by *M. tuberculosis* directly binds and activates STING molecules on the endoplasmic reticulum, thereby inducing a type I IFN immune response (Karanja et al., 2021). At the same time, the exogenous double-stranded DNA produced by *M. tuberculosis* is recognized by cyclic guanosine monophosphate/adenosine monophosphate synthase (cGAS) in host cells to generate endogenous cGAMP, which binds and activates the STING inducing a type I IFN immune response through transcription factor IRF3 (Cohen et al., 2019; Perez et al., 2022). Type I IFNs secreted from macrophages represents a major counter-regulatory class of inflammatory cytokines that control the outcome of *M. tuberculosis* infection (Ji et al., 2019). Additionally, autophagy is increasingly appreciated as a pivotal mechanism by which macrophages defend *M. tuberculosis* infection (Ning et al., 2021), and STING is able to induce autophagy (Moretti et al., 2017) through migrating from the endoplasmic reticulum to the Golgi during which the ER-Golgi intermediate compartment serves as a source of LC3-containing autophagosome membrane (Gui et al., 2019). Direct addition of c-di-AMP or DisA overexpressing strains of *M. tuberculosis* or BCG also induced autophagy in macrophages (Dey et al., 2015; Ning et al., 2021). In our study, unlike the *M. tuberculosis* infection process, CnpB was a foreign sterile stimulus that was possibly uptaken by macrophages through phagocytosis. As CnpB also degrades other cyclic di-nucleotides as a nanoRNase (Srivastav et al., 2014), reasonably CnpB negatively regulates the function of STING by degrading cGAMP rather than c-di-AMP, therefore inhibiting the autophagy in early stage in transcriptional level (**Figures 4C, D**) and LC3 protein levels in MH-S cells (**Figure 4F**).

We also analyzed NF-κB expression of MH-S after CnpB-stimulation by Western-blot, but no obvious changes were found (**Figure S3**). It has been reported that CnpB could degrade nanoRNAs (RNA oligos of ≤5 nucleotides) (Postic et al., 2012), and even hydrolyze c-di-GMP at a lower rate than it did on c-di-AMP (Bowman et al., 2016). In *S. aureus*, it was reported that the cytoplasmic DHH/DHHA1 phosphodiesterase PDE (Pde2) preferentially converts linear 5-phosphadenylyl-adenosine (pApA) to AMP (Woznica et al., 2021). Moreover, pApA is involved in a feedback inhibition loop that limits the membrane DHH/DHHA1 phosphodiesterase (GdpP) dependent c-di-AMP hydrolysis (Bowman et al., 2016). Bacteria must ensure control of c-di-AMP levels as both high levels and the absence of c-di-AMP result in several detrimental phenotypes. In eukaryotic cells, it was speculated that the transient upregulation of IFN-β induced by CnpB might be adjusted to normal by other nucleotides or unknown pathways, which was supported by the results of 24 h treatment (**Figure 4A**).

A study has also shown that CnpB could interfere with the cytosolic surveillance pathway of the host and the type I IFN response with *cnpB* mutant *M. tuberculosis* (Zhang et al., 2018). However, our results showed that CnpB protein upregulated IFN-β transcription in MH-S cells during the short time of treatment (**Figure 4A**), which was different from the IFN-β response induced by CnpB mutant strains (Zhang et al., 2018). The reason was very likely that degradation by antigen presentation process and reduced autophagy negatively regulated the cGAS-STING signaling pathway. In general, autophagy helps to prevent the host from producing excessive inflammatory cytokines (Zhang et al., 2021). The molecules involved in the autophagy pathway like ATGs can phosphorylate STING and suppresses IRF3 function to inhibit the transcription of IFN genes (Konno et al., 2013). And ubiquitination of cGAS and STING mediated by p62-dependent selective autophagic degradation was another inhibitory factor for type I IFN activation (Chen et al., 2016). Based on the above evidence, because of CnpB reducing autophagy and raising type I IFN in the early stages, CnpB treatment did not change the survival of *M. tuberculosis* either in MH-S or BMDM (**Figures 4G, H** **)**, and we inferred that post-infection treatment with CnpB had the same result. These suggested that the innate immune response induced by recombinant CnpB protein was insufficient to restrict bacterial survival in macrophages.

Infection models for evaluating vaccine efficacy include systemic infection (Moule and Cirillo, 2020) and pulmonary infection models (Plumlee et al., 2021). H37Ra is an attenuated strain of *M. tuberculosis*, often used for vaccine research (Bhavanam et al., 2020; Ning et al., 2021), and has also been used as a surrogate to study the virulence of *M. tuberculosis* with Biosafety Level 2 (BSL2) facilities (Heinrichs et al., 2018; Yang et al., 2020). In our previous work, mice were infected intravenously with attenuated strain H37Ra and virulent strain H37Rv, and we found that there were no significant differences in immune responses and bacteria burdens between the two strains within at least 8 w (Ning et al., 2017). Although studies have shown that macrophages infected with H37Ra and H37Rv respectively produced distinct cellular transcriptomic changes, (Pu et al., 2021), proteome levels of macrophages infected with H37Ra or H37Rv respectively showed very little expression difference in log and stationary phase, indicating H37Ra was able to induce a similar immune response with H37Rv (Wang et al., 2021). Thus, in this study the attenuated *M. tuberculosis* strain H37Ra was used instead of H37Rv to construct an intranasal infection model, which simulated the natural process of pulmonary infection similar to aerosol pathway (Logan et al., 2008). In previous work, we tested injecting a recombinant subunit vaccine directly into guinea pigs through an intravenous pathway, and found that it would cause severe systemic type I hypersensitivity reactions (Lu et al., 2016). Beforehand, we compared the immune responses induced by *M. tuberculosis* ESAT-6 with i.n. and s.c. (subcutaneous) vaccination, and found s.c. immunization of ESAT-6 with incomplete Freund’s adjuvant (IFA) could provide the highest humoral and cellular immune response (Lu et al., 2018). Thus, we chose s.c. vaccination with IFA in this study. As a result, the *M. tuberculosis* CFU in lungs and spleens reached about 10^4^ and 10^3^ per organ respectively after intranasal infection (**Figures 7C, D**). The pathology of the lungs of infected mice showed the infiltration of inflammatory cells and destroyed alveolar structure. CnpB subcutaneous immunization alleviated the pathological changes (**Figures 7A, B**), and reduced *M. tuberculosis* loads in lungs but not in spleens. This suggests that intranasal *M. tuberculosis* infection post CnpB subcutaneous immunization induced cellular and mucosal immune responses in lung, thus provided protection against *M. tuberculosis* infections in the respiratory tract.

Ag85B is the main protein secreted into the supernatant in the early stage of *M. tuberculosis* reproduction, and the secretion amount is the highest among Ag85 complex (Dheenadhayalan et al., 2002). Ag85B is currently the most studied and most immunogenic vaccine candidate antigen for *M. tuberculosis*. Ag85B-based recombinant DNA and recombinant rBCG vaccines have shown good protection against *M. tuberculosis* infection, could reduce the number of *M*. *tuberculosis* CFU in the spleens and lungs, and have entered clinical trials (Stylianou et al., 2015; Tkachuk et al., 2017; Jenum et al., 2021). It is interested for us to know whether CnpB has the potential to develop into a subunit vaccine-like Ag85B. In our previous study, we explored the cellular immune response induced by Ag85B and found that subcutaneous immunization of 50 μg Ag85B without IFA in mice increased the stimulation index of splenocyte proliferation by three folds (Lu et al., 2018). This study showed that subcutaneous immunization of CnpB with IFA adjuvant increased the stimulation index by four folds (**Figure 3B**). Meanwhile, our study found that humoral immune responses were induced by CnpB or Ag85B with IFA, but no significant difference between the two vaccines was observed (**Figure 2C**). Subcutaneous immunization with the CnpB provided protection against *M. tuberculosis* pulmonary infection in mice (**Figure 7C**). Concluding the above evidence, we speculated that CnpB could provide similar immune protection as Ag85B against *M. tuberculosis*. Next, we will try to combine the two proteins for the research of a subunit vaccine against *M*. *tuberculosis* infection.

In conclusion, this study provided evidence that CnpB had strong immunogenicity, inducing humoral and pulmonary cellular immune response after *M. tuberculosis* respiratory infection, which provided protection against *M. tuberculosis* infection. Thus, further work that the mechanism of CnpB regulating the innate immune cells and its application in vaccines against *M. tuberculosis* could be considered to investigate.

## Data Availability Statement

The original contributions presented in the study are included in the article/**Supplementary Material**. Further inquiries can be directed to the corresponding authors.

## Ethics Statement

The studies involving human participants were reviewed and approved by Institutional Ethics Committee of Second Affiliated Hospital of Air Force Medical University. The ethics committee waived the requirement of written informed consent for participation. The animal study was reviewed and approved by Institutional Ethics Committee of Second Affiliated Hospital of Air Force Medical University.

## Author Contributions

YL, HN, JK, LZ, YK, ZW, MT, and JZ performed the experiments. YL, HN, and JK analyzed the data. YL, YB, HN, GB, and YM wrote the manuscript. YB, YL, and YM conceived and designed the research. YB and YM supervised this work. All of the authors have read and agreed with the data. All authors contributed to the article and approved the submitted version.

## Funding

This study was funded by the National Major Special Projects of the 13th Five-year Plan (No. 2018ZX10302302002004), National Natural Science Foundation (No. 81971560, 81671638, 81371774), and the Provincial Natural Science Foundation of Shaanxi Province (2022ZDLSF01-07).

## Conflict of Interest

The authors declare that the research was conducted in the absence of any commercial or financial relationships that could be construed as a potential conflict of interest.

## Publisher’s Note

All claims expressed in this article are solely those of the authors and do not necessarily represent those of their affiliated organizations, or those of the publisher, the editors and the reviewers. Any product that may be evaluated in this article, or claim that may be made by its manufacturer, is not guaranteed or endorsed by the publisher.
