# Supplementary material for: Cyclic-di-AMP Phosphodiesterase Elicits Protective Immune Responses Against Mycobacterium tuberculosis H37Ra Infection in Mice
Source: Front Cell Infect Microbiol. 2022 Jun 22;12:871135. doi: 10.3389/fcimb.2022.871135 (PMC9256937; doi:10.3389/fcimb.2022.871135)
Supplement: Supplementary file 1 [file DataSheet_1.pdf]

Table S1 Primers used in this study

Genes	Primers
CnpB	5’ -CGGCATATGGTGACGACGATCGACCCAAG- 3’
	5’ -TTTAAGCTTGCCAGCTGCGCGATCTGACG- 3’
CnpB-DHH	5’ -CGCCATATGTCGTGCTGGGAAATGAAG- 3’
(1-179aa)	5’ -ATTGGATCCGCAGTAGGGCTGCAGTTTG- 3’
CnpB-DHHA1	5’ -TTTCCATGGACACGGGGTCGTTTCGC- 3’
(180-336aa)	5’ -TTTAAGCTTACCAAGCGCCGCGCGCAAC- 3’
Mouse IFN-γ	5’ -CATTGAAAGCCTAGAAAGTCTGAATAAC- 3’
	5’ -TGGCTCTGCAGGATTTTCATG- 3’
Mouse IL-10	5’ -GAAGACCCTCAGGATGCGG- 3’
	5’ -ACCTGCTCCACTGCCTTGCT- 3’.
Mouse IFN-β	5’ -CATTGAAAGCCTAGAAAGTCTGAATAAC- 3’
	5’ - TGGCTCTGCAGGATTTTCATG- 3’
Mouse Atg5	5’ -TGTGCTTCGAGATGTGTGGTT- 3’
	5’ -ACCAACGTCAAATAGCTGACTC- 3’
Mouse Atg7	5’ -TCTGGGAAGCCATAAAGTCAGG- 3’
	5’ -GCGAAGGTCAGGAGCAGAA- 3’
Mouse GAPDH	5’ -AGCTTCGGCACATATTTCATCTG- 3’
	5’ -CGTTCACTCCCATGACAAACA- 3’

Table S2 Antigenicity and allergenecity prediction of CnpB and other antigens

Proteins	Vaxi-Jen	AllerTOP
CnpB	0.5891 (Probable Antigen)	Probable non-allergen
Ag85B	0.5842 (Probable Antigen)	Probable non-allergen
ESAT-6	0.5577 (Probable Antigen)	Probable non-allergen

Table S3 B-cell and T-cell epitopes prediction of CnpB

B-cell epitopes prediction

**Server**	**$_{Position}$Sequence**
IEDB	$_{5}$<u>DPRSELVDGRRRAGA</u>
IEDB	$_{75}$TLPESLRSLPGCHLLVRPEVMRRD
IEDB	$_{109}$SVDRLGALGDLTD
IEDB	$_{184}$SFRWASVR
IEDB	$_{203}$<u>GVDNATVSR</u>
IEDB	$_{214}$MDSHPFTW
IEDB	$_{233}$QLVSEAV
IEDB	$_{251}$<u>NREWVAARSEEVES</u>
IEDB	$_{309}$<u>GGGHR</u>
BCPREDS	$_{2}$TTI<u>DPRSELVDGRRRAGA</u>RV
BCPREDS	$_{138}$FGTANFIDPSADSTTTMVAE
BCPREDS	$_{159}$LDAWGKPIDPRVAHCIYAGL
BCPREDS	$_{202}$I<u>GVDNATVSR</u>TLMDSHPFTW
BCPREDS	$_{247}$VVVD<u>NREWVAARSEEVES</u>IV
BCPREDS	$_{306}$GFG<u>GGGHR</u>LAAGYTTTGSID

T-cell epitopes prediction

**Server**	**$_{Position}$Sequence**
NetCTL	$_{3}$TIDPRSELV
NetCTL	$_{119}$LTDSGRELL
NetCTL	$_{147}$SADSTTTMV
NetCTL	$_{179}$ATDTGSFRW
NetCTL	$_{213}$LM<u>DSHPFTW</u>
NetCTL	$_{238}$AVGGRGLVY
NetMHC	$_{27}$ALLSAAARV
NetMHC	$_{193}$RLAARLVEI
NetMHCII	$_{75}$TLPESLRSLPGCHLLVRPE
NetMHCII	$_{215}$<u>DSHPFTW</u>LPLLSRVL GSAQ
NetMHCII	$_{285}$EPHRWSVSMRAKTVNLAAV

Note: the underline sequences refer to sequences predicted at least twice.

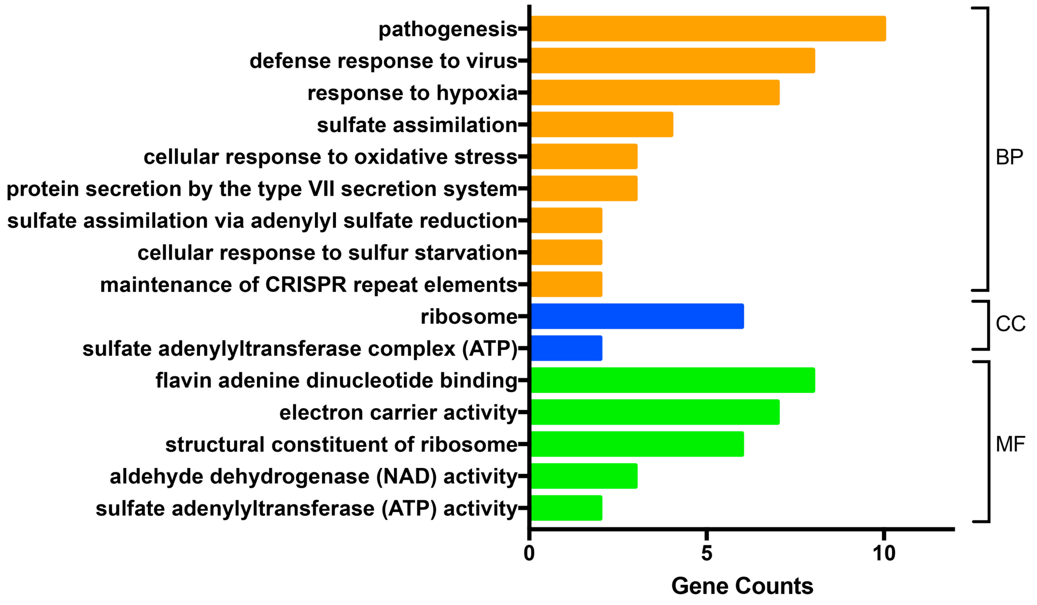

Figure S1. Immunoinformatics analysis of CnpB of Mtb. Gene Ontology (GO) analysis of transcriptomic profile in MtbΔCnpB from GSE102816 of GEO database.

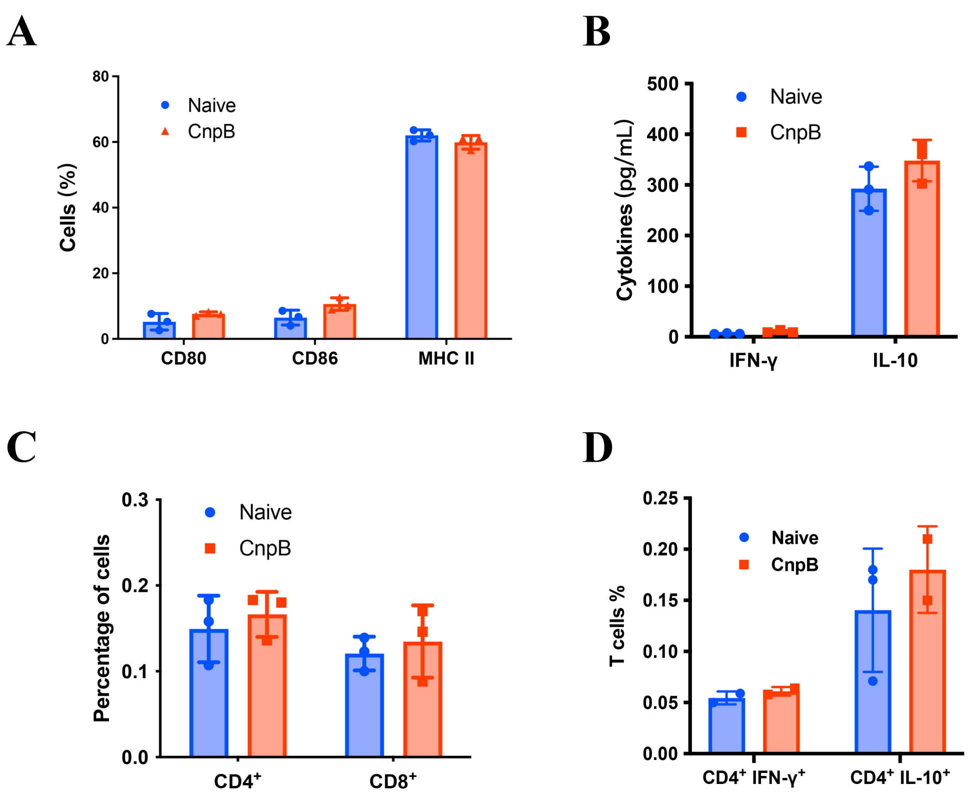

Figure S2. **Cellular immune response in splenocytes of CnpB-immunized mice.** (**A**) Proportions of T cells in the spleens of CnpB immunized mice and naive mice by flow cytometry. (**B**) Detection of the levels of Th1 (IFN-γ) and Th2 (IL-10) cytokines produced by splenocytes of CnpB-immunized mice by ELISA. (**C**) Flow cytometry was used to detect CD4$^+$ and CD8$^+$ T cells of splenocytes from CnpB-immunized mice. (**D**) Flow cytometry detected IFN-γ or IL-10 produced CD4$^+$ T cells in spleens of CnpB-immunized mice, and the proportion of CD4$^+$ IFN-γ$^+$ T (Th1) cells and CD4$^+$ IL-10$^+$ T (Th2) cells were analyzed. These cells were stimulated by 5 μg/mL CnpB proteins. The results are expressed as the mean ± SD.

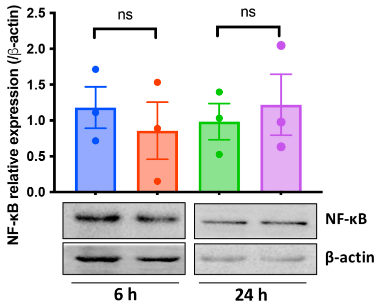

Figure S3. **NF-κB expression in macrophages after CnpB treatment**. MH-S cells were treated with CnpB for 6 h and 24 h, respectively. NF-κB expression was analyzed by Western blot. The results are expressed as the mean ± SD.

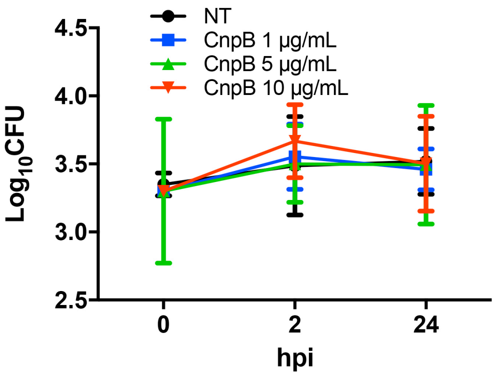

Figure S4. **Intracellular survival of *M. tuberculosis* in macrophages.** MH-S cells were treated with different concentrations CnpB for 6 h, then cells were infected with *M. tuberculosis* H37Ra at MOI of 2. Bacteria were removed post 4 h infection, this time point termed as “0 h”. Bacteria CFUs were determined at indicated time points post infection.

Supplemental Method

Bioinformatics Analysis

Search for 'CnpB' in GEO database (https://www.ncbi.nlm.nih.gov/gds) in NCBI, and download the GSE102816 data containing transcriptome sequencing of *M. tuberculosis* knockout CnpB. The –log10(FC) threshold was set as ± 0.58496250072, and differential expression genes over ± 1.5 fold change were screened out. Then, the gene ontology (GO) analysis of differentially expressed genes were performed through DAVID (https://david.ncifcrf.gov) online tool.
